# Juvenile Hormone-Sensitive Ribosomal Activity Enhances Viral Replication in Aedes aegypti

**DOI:** 10.1128/mSystems.01190-20

**Published:** 2021-05-26

**Authors:** Zuo-Kun Shi, Dan Wen, Meng-Meng Chang, Xiao-Mei Sun, Yan-Hong Wang, Chi-Hang Cheng, Li-Qin Zhang, Ai-Hua Zheng, Zhen Zou

**Affiliations:** a State Key Laboratory of Integrated Management of Pest Insects and Rodents, Institute of Zoology, Chinese Academy of Sciences, Beijing, China; b CAS Center for Excellence in Biotic Interactions, University of Chinese Academy of Sciences, Beijing, China; c Key Laboratory of Vector Biology and Pathogen Control of Zhejiang Province, Huzhou University, Huzhou, China; University of California, San Diego

**Keywords:** mosquito, antiviral, LRIM, ribosome, juvenile hormone

## Abstract

Zika virus (ZIKV; *Flaviviridae*) is a devastating virus transmitted to humans by the mosquito Aedes aegypti. The interaction of the virus with the mosquito vector is poorly known. The double-stranded RNA (dsRNA)-mediated interruption or activation of immunity-related genes in the Toll, IMD, JAK-STAT, and short interfering RNA (siRNA) pathways did not affect ZIKV infection in A. aegypti. Transcriptome-based analysis indicated that most immunity-related genes were upregulated in response to ZIKV infection, including leucine-rich immune protein (LRIM) genes. Further, there was a significant increment in the ZIKV load in *LRIM9-*, *LRIM10A-*, and *LIRM10B*-silenced A. aegypti, suggesting their function in modulating viral infection. Further, gene function enrichment analysis revealed that viral infection increased global ribosomal activity. Silencing of *RpL23* and *RpL27*, two ribosomal large subunit genes, increased mosquito resistance to ZIKV infection. *In vitro* fat body culture assay revealed that the expression of *RpL23* and *RpL27* was responsive to the Juvenile hormone (JH) signaling pathway. These two genes were transcriptionally regulated by JH and its receptor methoprene-tolerant (Met) complex. Silencing of *Met* also inhibited ZIKV infection in A. aegypti. This suggests that ZIKV enhances ribosomal activity through JH regulation to promote infection in mosquitoes. Together, these data reveal A. aegypti immune responses to ZIKV and suggest a control strategy that reduces ZIKV transmission by modulating host factors.

**IMPORTANCE** Most flaviviruses are transmitted between hosts by arthropod vectors such as mosquitoes. Since therapeutics or vaccines are lacking for most mosquito-borne diseases, reducing the mosquito vector competence is an effective way to decrease disease burden. We used high-throughput sequencing technology to study the interaction between mosquito Aedes aegypti and ZIKV. Leucine-rich immune protein (LRIM) genes were involved in the defense in response to viral infection. In addition, RNA interference (RNAi) silencing of *RpL23* and *RpL27*, two JH-regulated ribosomal large subunit genes, suppressed ZIKV infection in A. aegypti. These results suggest a novel control strategy that could block the transmission of ZIKV.

## INTRODUCTION

Zika virus (ZIKV) is a mosquito-borne virus of the genus *Flavivirus*, which includes many other human pathogens, such as yellow fever virus (YFV), West Nile virus (WNV), Japanese encephalitis virus (JEV), and dengue virus (DENV). Since 2007, ZIKV has caused epidemics in French Polynesia, the South Pacific, and, more recently, in South and North America (1–3). The clinical symptoms are variable and range from asymptomatic, mild symptoms to severe neurological complications, such as Guillain-Barré syndrome in adults or microcephaly in neonates (4–6). After the mosquito acquires the virus through a blood feeding from the infected host, the virus infects the intestinal epithelial cells and systematically spreads to other tissues, such as the neural system, fat bodies, and salivary glands. The infected mosquitoes then can transmit the virus to other hosts through blood feeding (7–9). Since therapeutics and vaccines are unavailable for most mosquito-borne diseases, a better understanding of mosquito-virus interactions might provide new ideas for targeting viral transmission and decreasing the disease burden.

Once mosquitoes are infected with the virus, they will be infected for life and carry a high viral load without harmful effects (10). This reflects the coevolution between mosquitoes and viruses. Mosquitoes possess multiple immune mechanisms that can eliminate or restrict infection (11, 12). Viruses have developed diverse mechanisms to subvert or evade host defenses and change the tissue environment or host cells to promote infection. For example, flaviviruses utilize their own nonstructural protein 1 (NS1), secreted into the host blood, to efficiently overcome the mosquito gut barrier and enhance infection (13, 14). The complete flavivirus life cycle requires the use of many host factors. Flavivirus enters the cell through endocytosis initiated when the viral particle interacts with the cell surface receptor, which mainly include heat shock proteins, C-type lectin (mosGCTL-1), and laminin receptors in the mosquito cells (15–18). Upon entry, the viral genomes are translated, replicated, and packaged into virions in the membrane compartments associated with the endoplasmic reticulum (ER). They then develop into infectious particles and are released from the cell by secretion (19). These processes require the involvement of a variety of host cellular factors to work for the virus (e.g., ribosomal elements) (19, 20).

The RNA interference (RNAi) pathway is an essential antiviral mechanism in Drosophila melanogaster (21–23). Once challenged by viruses, Dicer2 acts as a pattern recognition receptor (PRR), which binds to virus-derived double-stranded RNA (dsRNA) and cleaves it into short interfering RNAs (siRNAs) (22). The virus-derived siRNA (vsiRNA) then is used as a guide strand to specifically degrade viral RNA through the RNase activity of Argonaute-2 (Ago2) (21). vsiRNAs have been detected in *Aedes* mosquito cell infection with arboviruses (24, 25). Knockdown of *Dicer2* or *Ago2* increases the replication of arbovirus in mosquitoes and improves the efficiency of arbovirus transmission (26, 27).

The Janus kinase/signal transducers and activators of transcription (JAK-STAT) pathway is an evolutionarily conserved pathway that induces antiviral effectors in mammals and arthropods (28, 29). Loss of function of the Janus kinase (Hop) and receptor Domeless (Dome) increases the DENV burden, while the resistance to DENV infection is enhanced by silencing *PIAS* (a negative regulator of the JAK-STAT pathway) (28). In addition, the induction of interferon-like cytokine Vago restricted WNV replication in Culex quinquefasciatus Hsu cells by triggering the JAK-STAT signaling cascade (30).

Toll and immune deficiency (IMD) are two other important immune signaling pathways in mosquitoes and other insects. Immune activation enables NF-κB factors to enter the nucleus and transcriptionally initiate downstream gene expression, including antimicrobial peptides (AMPs) (31). DENV infection induces activation of both pathways in A. aegypti (32). A. aegypti infection with DENV is increased by knockdown of the components of these pathways (33, 34).

The insect-specific steroid hormone, 20-hydroxyecdysone (20E), and the sesquiterpenoid hormone, juvenile hormone (JH), are the two major hormones that control the development and reproduction of mosquitoes. Research on mating and reproductive physiology has led to the development of sterilizing compounds for mosquitoes based on JH and 20E (35–38). The JH analogue pyriproxyfen (PPF) and the 20E agonist methoxyfenozide (DBH) cause sterility and reduce the life span of adult mosquitoes (39, 40). However, the interaction of these hormones with pathogens has rarely been reported. Although DBH can reduce *Plasmodium* parasite development in *Anopheles* mosquitos by an unknown mechanism (40), it is unknown if these molecules can affect viral infections.

Previously, we have shown that the induction of *HPX8C*, an antioxidant gene, enhances DENV infection through removal of reactive oxygen species (ROS) in the mosquito midgut (41). ZIKV envelope glycosylation modification also can specifically inhibit the ROS pathway in the mosquito midgut and successfully cross the midgut barrier (42). These findings suggested that the ROS pathway has an antiviral role in mosquitoes. In this study, we studied the reaction of mosquitoes to ZIKV infection by examining the tissue-specific transcriptional response of A. aegypti to ZIKV infection and tried to find out more immune and physiological restriction factors in limiting virus infection.

## RESULTS

### Classical antiviral immune pathways do not affect ZIKV infectivity in A. aegypti.

**FIG 1:**
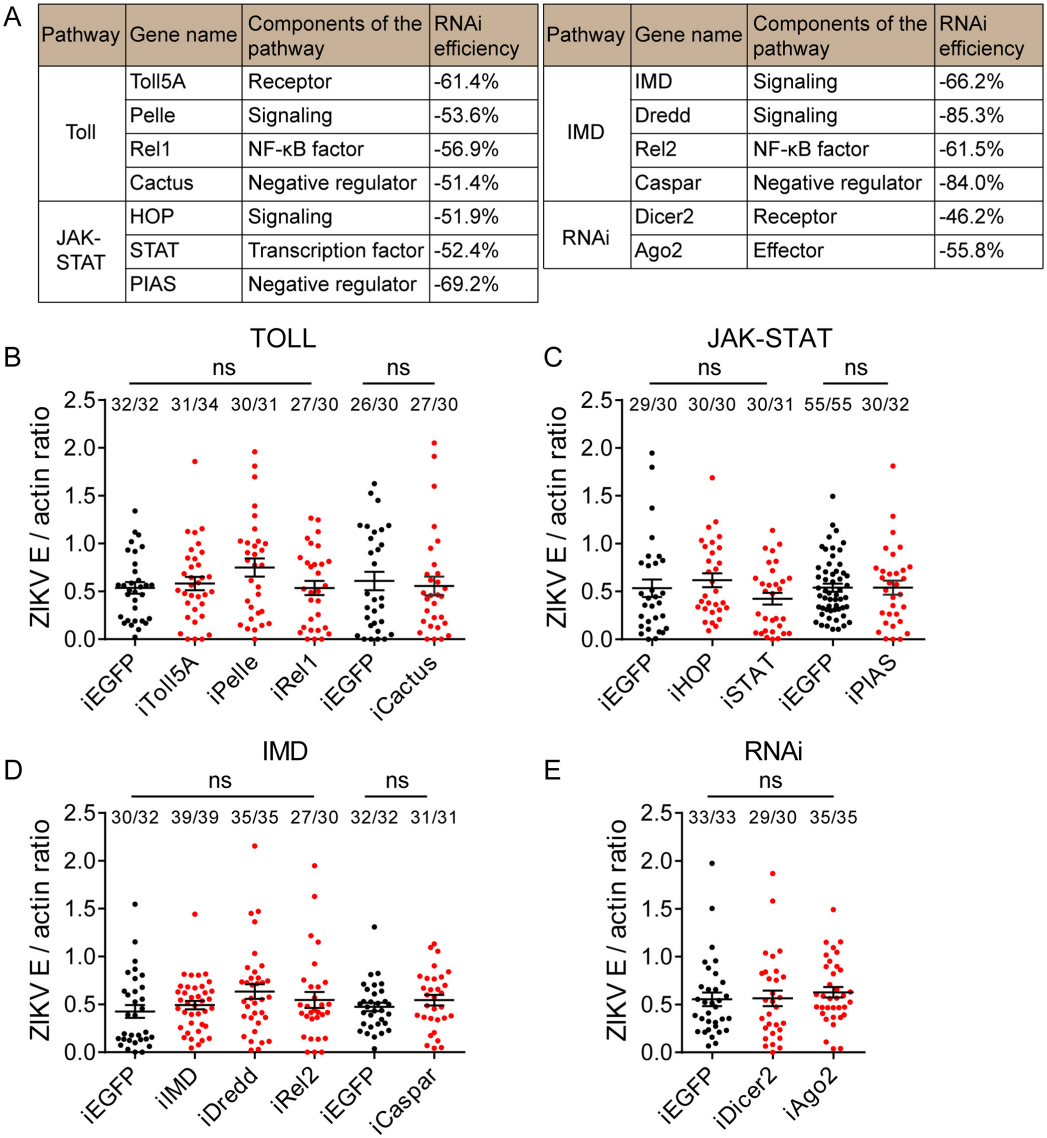
Toll, JAK-STAT, IMD, and RNAi pathways do not affect ZIKV infectivity in A. aegypti. (A) Merged list of selected genes for RNAi screening research. The RNAi efficiency of 5 to 10 mosquitoes was measured at 7 dpi, shown as means from 2 to 5 replicates. (B to E) dsRNA was microinjected into the thorax of 1-day-old female mosquitoes. After a 3-day recovery period, these females were fed blood meal containing 1.0 × 10^6^ PFU/ml ZIKV. At 7 dpi, total RNA of a single mosquito was isolated, and the viral load was determined by qPCR. The viral load was normalized against the A. aegypti actin gene. *EGFP* dsRNA-treated mosquitoes were the control group. The top of each column shows the ratio of the number of infected mosquitoes to the total number of mosquitoes. Data are means ± SEM. The result shown is pooled from two independent experiments. A Mann-Whitney test was used for the statistical analysis. ns, not significant.

Toll, IMD, JAK-STAT, and RNAi are important antiviral defense pathways in mosquitoes, but most studies have focused on the role of anti-DENV (25, 28, 33, 34, 43), and little is known about their relationship with ZIKV. To investigate the effect of these immune pathways on ZIKV infection, RNAi screening studies were conducted. We selected 13 genes from Toll, IMD, JAK-STAT, and RNAi pathways for analysis (Fig. 1A). Compared to the *EGFP* dsRNA-injected group, the selected genes were still silenced by 46% to 85% at 7 days postinfection (dpi) (Fig. 1A). A. aegypti infection with ZIKV was not enhanced by knocking down key elements of the Toll, IMD, and JAK-STAT pathway, which included genes *Toll5A*, *Pelle*, *Rel1*, *IMD*, *Dredd*, *Rel2*, *HOP*, and *STAT* (Fig. 1B to D). Cactus, Caspar, and PIAS are negative regulators of the Toll, IMD, and JAK-STAT pathway, respectively. Knockdown of these genes can activate the immune pathway and promote the expression of downstream effectors, such as AMPs (28, 44). Silencing of *Cactus*, *Caspar*, and *PIAS* did not increase resistance to ZIKV infection (Fig. 1B to D). In addition, the depletion of the RNAi pathway genes *Dicer 2* and *Ago2* also did not enhance ZIKV infection (Fig. 1E). These results indicate that these pathways do not have an anti-ZIKV role in A. aegypti.

### The immune response to ZIKV infection in A. aegypti.

To examine the immune response of A. aegypti against ZIKV infection more systematically, we performed a global transcriptome analysis on the midgut (the first barrier of virus infection) and fat body (the main immune tissue of mosquitoes) after blood feeding. A blood meal containing 1.0 × 10^6^ PFU/ml of ZIKV diluted in mouse blood and 50% RPMI 1640 medium was fed to A. aegypti. At 1, 4, 7, and 10 dpi, total RNA was isolated from the midguts and fat bodies, and viral RNA was analyzed by quantitative reverse transcription-PCR (qPCR). After oral infection, ZIKV rapidly replicated in the midgut. The midgut viral RNA increased by about 1,000-fold at 4 dpi compared with 1 dpi and then reached about 3,000-fold at 10 dpi (see Fig. S1A in the supplemental material). ZIKV was readily detectable at 7 dpi in fat bodies (Fig. S1A).

10.1128/mSystems.01190-20.1 FIG S1 (A and B) 3- to 4-day-old female A. aegypti was fed blood meal containing 1.0 × 10^6^ PFU/ml ZIKV. Total RNA was isolated from 30 midguts or 30 fat bodies at the time points shown. (A) ZIKV replication in the midgut and fat body. The viral load was tested by qPCR and normalized against the A. aegypti actin gene. Data were normalized with the viral load of 1 dpi midgut. Data are means ± SEM from three independent experiments. (C) Venn diagram analysis of DEGs in fat body (left) and midgut (right) after ZIKV infection. DEGs were generated from the comparison between the infected group and the mock group at each time point. The DEGs with a *P* value of <0.01 and a ratio of >1.5-fold were selected for further analysis. The direction of gene changes is indicated by up and down arrows. Fb_1d, fat body at 1 dpi; Fb_7d, fat body at 7 dpi; Mg_1d, midgut at 1 dpi; Mg_7d, midgut at 7 dpi. Download FIG S1, TIF file, 0.5 MB. Copyright © 2021 Shi et al. 2021 Shi et al. https://creativecommons.org/licenses/by/4.0/ This content is distributed under the terms of the Creative Commons Attribution 4.0 International license.

Transcriptomes from the midguts and fat bodies of uninfected and ZIKV-infected mosquitoes at 1 and 7 dpi were generated by paired-end sequencing on the Illumina RNA sequencing (RNA-Seq) platform. Differentially expressed gene (DEG) analysis was performed using the edgeR package. Upon infection, 2,766 DEGs were upregulated or downregulated, with a change of more than 1.5 times (Table S1). The number of altered genes in the fat body (1,069 DEGs at 1 dpi, 1,796 DEGs at 7 dpi) was more than that in the midgut (573 DEGs at 1 dpi, 308 DEGs at 7 dpi) (Fig. S1B). Among these DEGs, 430 (75%, 1 dpi) and 285 (93%, 7 dpi) genes were significantly upregulated in the midgut, while 856 (80%, 1 dpi) and 1,282 (71%, 7 dpi) genes were significantly downregulated in the fat body (Fig. S1B). These results indicate that A. aegypti has a substantial tissue-specific response to ZIKV infection. This is consistent with the results of a previous report on the response of A. aegypti to DENV infection (41).

10.1128/mSystems.01190-20.8 TABLE S1 Whole list of DEGs identified in A. aegypti midgut and fat body transcript analysis. Download Table S1, XLSX file, 0.6 MB. Copyright © 2021 Shi et al. 2021 Shi et al. https://creativecommons.org/licenses/by/4.0/ This content is distributed under the terms of the Creative Commons Attribution 4.0 International license.

**FIG 2:**
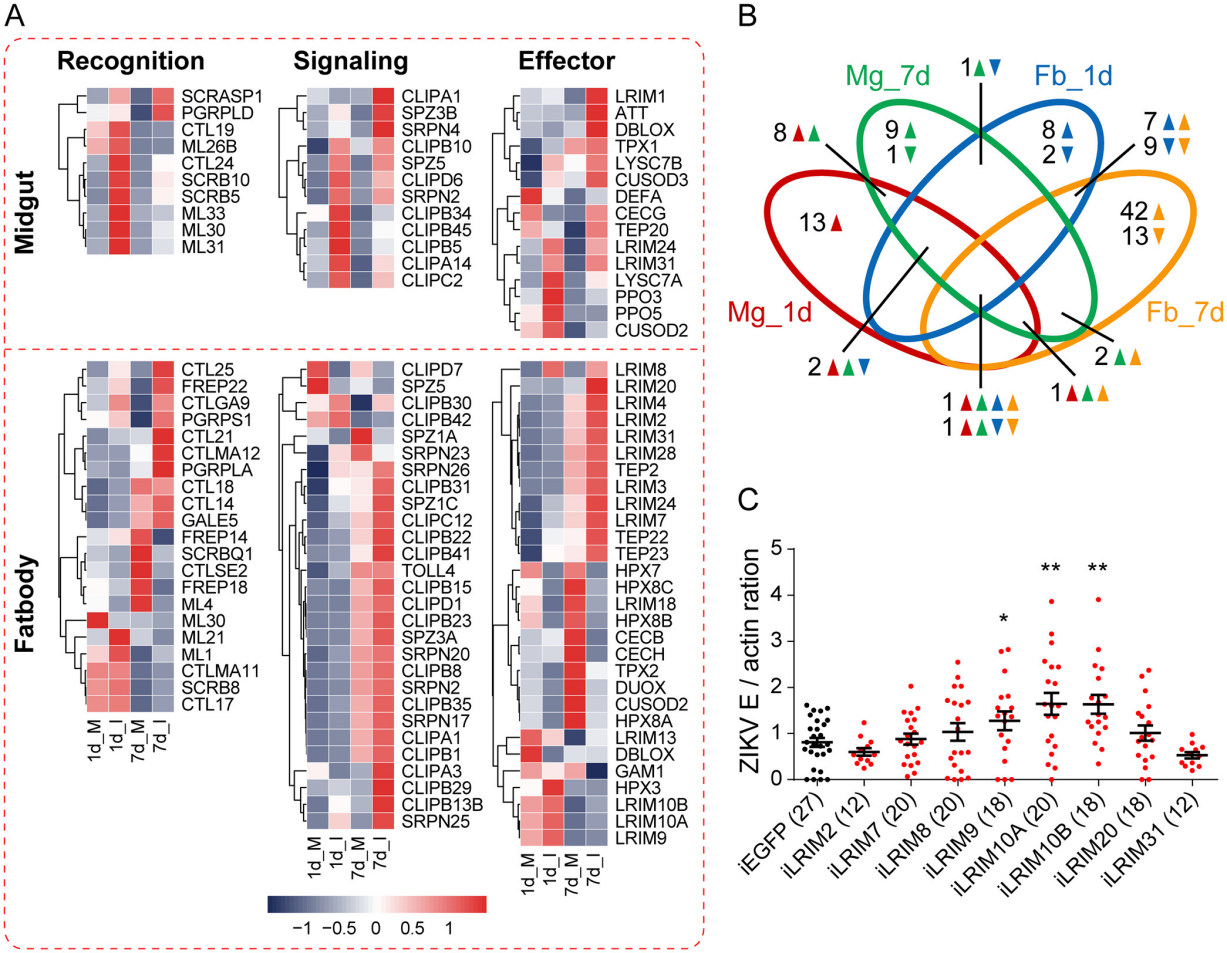
Immunity-related gene expression in the midgut and fat body are distinct. (A) Heatmap analysis of immunity-related DEGs following viral infection. According to their functions, immune genes were divided into three categories: recognition molecules, signaling regulators, and immune effectors. The FPKM values from three replicates were used to plot the heatmap. (B) Venn diagram analysis of immunity-related DEGs that are regulated by ZIKV infection in two tissues. The direction of gene changes is indicated by up and down arrows. Mg, midgut; Fb, fat body. (C) LRIMs are involved in the anti-ZIKV defense. After gene silencing, female A. aegypti organisms were fed blood meal containing 1.0 × 10^6^ PFU/ml ZIKV. At 7 dpi, the ZIKV viral load was tested by qPCR and normalized against the A. aegypti actin gene. *EGFP* dsRNA-treated females were the control group. One dot represents 1 mosquito, and the number of mosquitoes used for testing is shown in brackets. The experiment was repeated three times with similar results. Data are means ± SEM, and the *P* value was determined by a Mann-Whitney test. *, *P < *0.05; **, *P < *0.01.

We then focused on the expression profiles of immunity-related genes. The annotation of the immunity-related gene is assigned based on immunedb, a database containing information about insect immunity-related genes and gene families (45). Hierarchical clustering revealed the expression profiles of immune DEGs in different tissues, including the molecules involved in pattern recognition, signal modulation, and execution (Fig. 2A). In the midgut, 39 immune transcripts were differentially expressed, of which 38 were upregulated and only 1 was downregulated by viral infection (Fig. 2B). In the fat body, 88 immune genes were regulated by viral infection, 61 of which were upregulated and 27 downregulated. Among these genes, only 18 were common both at 1 and 7 dpi (Fig. 2B, Table S2). In detail, 25 immune recognition molecules were significantly upregulated in the midgut and fat body, mainly including 10 C-type lectins (CTLs), 6 MD2-like receptors (MLs), 4 scavenger receptors (SCRs), and 3 peptidoglycan recognition proteins (PGRPs). Of the 73 clip-domain serine protease (CLIP) genes involved in the signal cascade, 23 were upregulated due to viral infection. Seven transcripts encoding Serpin (SRPNs) were induced by viral infection either in the midgut or fat body. In the midgut, most immune effectors were activated, including antimicrobial peptides (*DefA*, *Att*, and *CecG*), prophenoloxidases (*PPO3* and *PPO5*), and superoxide dismutases (*CuSOD2* and *CuSOD3*). In the fat body, 13 leucine-rich immune protein (LRIM) genes were induced at 7 dpi. Together, these upregulated immunity-related genes may have roles in ZIKV infection.

10.1128/mSystems.01190-20.9 TABLE S2 DEGs of immunity and translation element identified in A. aegypti midgut and fat body transcripts analysis. Download Table S2, XLSX file, 0.04 MB. Copyright © 2021 Shi et al. 2021 Shi et al. https://creativecommons.org/licenses/by/4.0/ This content is distributed under the terms of the Creative Commons Attribution 4.0 International license.

Next, we selected LRIMs to verify the immunotranscriptome of A. aegypti in response to ZIKV infection. LRIM immunity was first reported in *Anopheles* mosquitoes against malaria (46, 47). We validated these upregulated genes in the fat body at 7 dpi using qPCR and found 9 *LRIM* genes were induced 1.2- to 1.5-fold after infection, including *LRIM2*, *LRIM7*, *LRIM8*, *LRIM9*, *LRIM10A*, *LRIM10B*, *LRIM20*, *LRIM24*, and *LRIM31* (Fig. S2A). Next, dsRNA-mediated gene silencing studies assessed the role of LRIM in ZIKV infection of A. aegypti. Efficiency of RNAi knockdown was confirmed using qPCR (Fig. S2B). The ZIKV load was unaffected by *LRIM2*, *LIRM7*, *LRIM8*, *LRIM20*, and *LRIM31* silencing (Fig. 2C). In contrast, there were 1.6-, 2.0-, and 2.0-fold increments in the ZIKV loads in *LRIM9-*, *LRIM10A-*, and *LIRM10B*-silenced A. aegypti compared to controls (Fig. 2C), suggesting that these genes modulate ZIKV infection in mosquitoes.

10.1128/mSystems.01190-20.2 FIG S2 (A) Expression of LRIM genes in the fat body after ZIKV infection. At 7 dpi, the expression of *LRIMs* in 30 fat bodies was tested by qPCR and normalized against the A. aegypti actin. Data were normalized with the mRNA level of the mock group, indicated by the red dotted line, and shown as mean ± SEM (*n* = 3). *P* value was determined by unpaired Student’s *t* tests. *, *P < *0.05; **, *P < *0.01. (B) RNAi efficiency of LRIM genes at 7 dpi (at 10 days after dsRNA treatment). The gene expression was tested by qPCR and normalized against A. aegypti actin. *EGFP* dsRNA-treated mosquitoes were the control group. Data are means ± SEM (*n* ≥ 3). Download FIG S2, TIF file, 0.5 MB. Copyright © 2021 Shi et al. 2021 Shi et al. https://creativecommons.org/licenses/by/4.0/ This content is distributed under the terms of the Creative Commons Attribution 4.0 International license.

### Ribosomal component proteins are the critical host factors for ZIKV infection.

**FIG 3:**
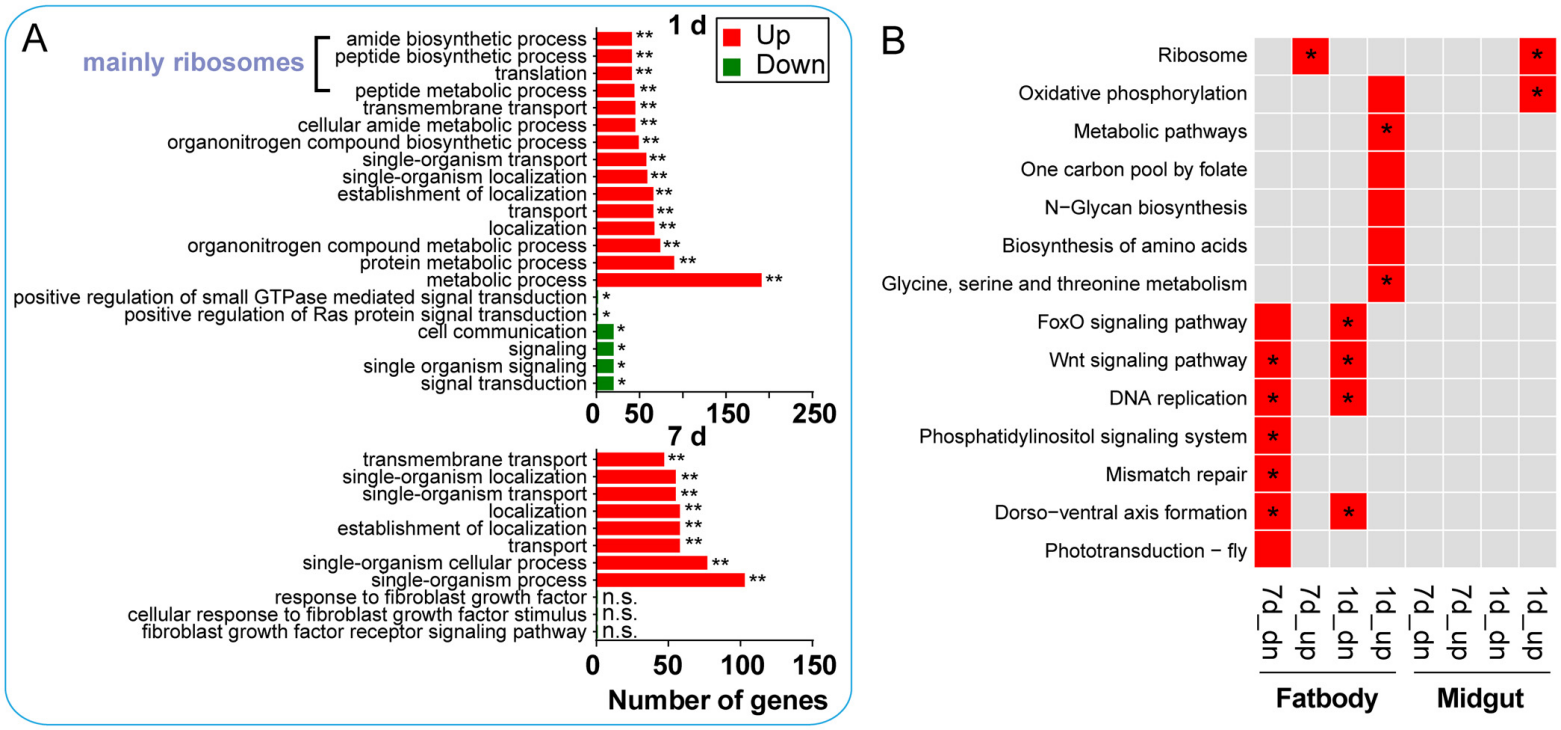
Functional classification of the different expression genes. (A) GO enrichment analysis of midgut transcripts significantly regulated by ZIKV infection. The GO terms corresponding to biological process were analyzed. The GO terms most associated with the upregulated and downregulated enriched genes are shown. Statistical significance was determined at the false discovery rate. *, adjusted *P* value (*P*_adj_), <0.05; **, *P*_adj_ < 0.01; ns, not significant. (B) KEGG functional classification of midgut and fat body transcripts significantly regulated by ZIKV infection. The KEGG pathways most associated with the upregulated and downregulated enriched genes are shown in the red box (*P*_adj_ < 0.05) or in the red box with an asterisk (*P*_adj_ < 0.01). Statistical significance was determined at the false discovery rate. Up, upregulated genes; dn, downregulated genes.

Apart from immunity, the interaction between vector and virus also involves many other physiological factors. GO (gene ontology) and KEGG (Kyoto Encyclopedia of Genes and Genomes) are databases for gene function annotation based on different classification methods. Therefore, functional pathway enrichment analysis of DEGs was performed to identify other potential essential restriction factors involved in limiting or promoting viral infection. Strikingly, both analyses indicated that ribosomal protein genes were affected by viral infection. Although the midgut and fat body are significantly different in response to viral infections, many protein synthesis-related GO terms and ribosomal protein genes were enriched in the upregulated genome of the midgut 1 dpi and fat body 7 dpi (Fig. 3, Fig. S3).

10.1128/mSystems.01190-20.3 FIG S3 GO enrichment analysis of DEGs in fat body. The GO terms corresponding to biological process were analyzed. The GO terms most associated with the upregulated and downregulated enriched genes are shown. Statistical significance was determined at the false discovery rate. *, *P*_adj_ < 0.05; **, *P*_adj_ < 0.01. Download FIG S3, TIF file, 1.2 MB. Copyright © 2021 Shi et al. 2021 Shi et al. https://creativecommons.org/licenses/by/4.0/ This content is distributed under the terms of the Creative Commons Attribution 4.0 International license.

**FIG 4:**
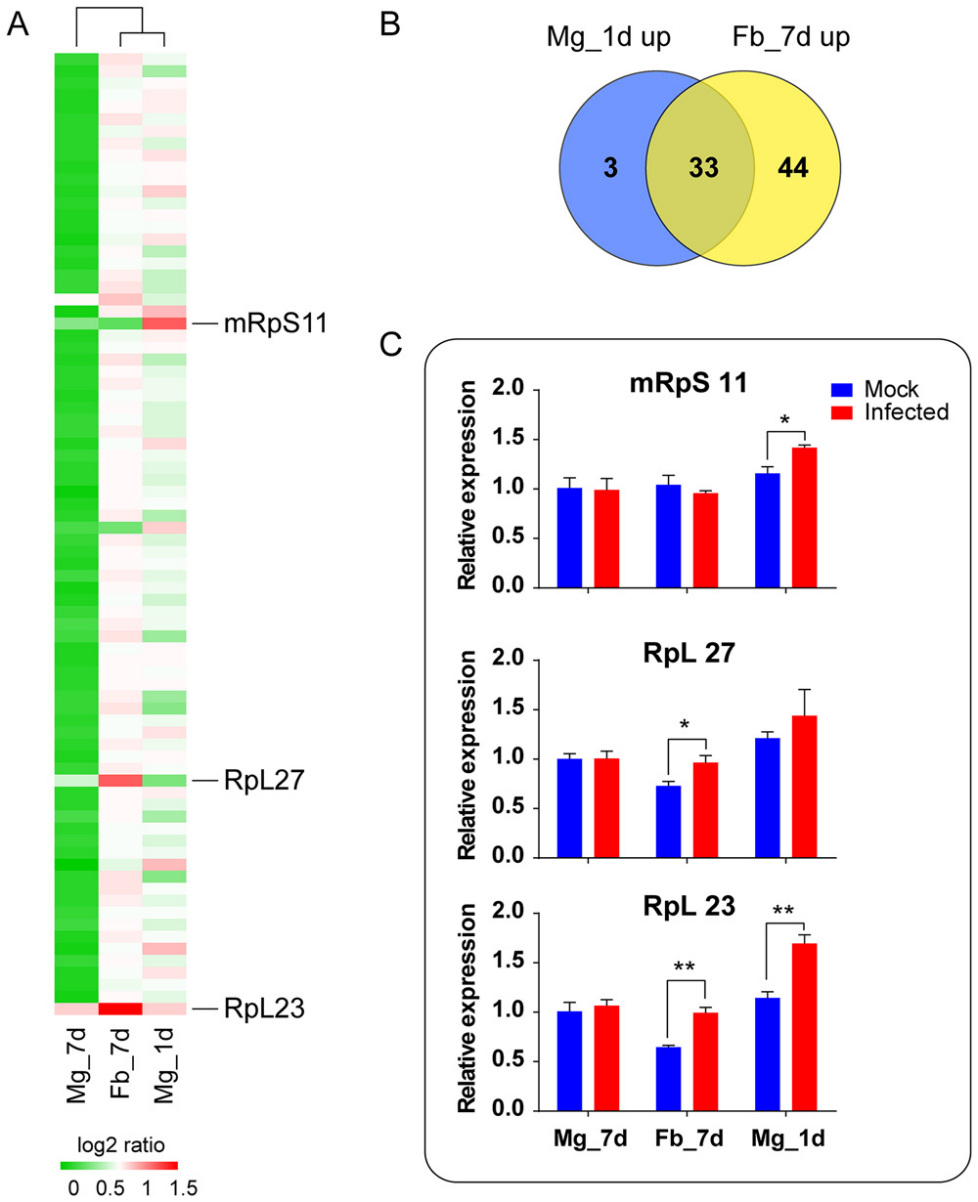
Global ribosome genes are induced after early infection. (A and B) The upregulated ribosomal protein transcripts from 1-dpi midgut and 7-dpi fat body were collected for analysis. (A) Heatmap analysis revealed the induction of ribosomal protein transcripts after viral infection. The corresponding 7-dpi midgut transcripts were the control. The log_2_ ratio (read number in the virus-infected group/read number in the mock group) was used to plot the heatmap. Mg, midgut; Fb, fat body. (B) Venn diagram analysis of ribosomal protein genes significantly induced by ZIKV infection. Mg_1 d_up, upregulated genes from 1-dpi midgut; Fb_7 d_up, upregulated genes from 7-dpi fat body. (C) qPCR analysis of selected ribosome-related genes. Unpaired Student’s *t* tests were used for statistical analysis. The result shown is the mean ± SEM from three independent experiments. *, *P < *0.05; **, *P < *0.01.

In further analysis, most ribosomal protein genes (80 out of 109) were upregulated from 1.5-fold to 2.8-fold in the midgut or fat body after ZIKV infection (Table S2). These genes were induced only in the midgut 1 dpi or in the fat body 7 dpi (Fig. 4A). The Venn diagram (Fig. 4B) indicated that 33 (about 41% of the total) genes were common to these two infection groups, and 3 and 44 were unique to the midgut 1 dpi and the fat body 7 dpi, respectively. We chose three top DEGs to perform qPCR analysis, *mRpS11*, *RpL23*, and *RpL27*, marked in Fig. 4A. Upon infection, the expression of *mRpS11*, *RpL23*, and *RpL27* was significantly upregulated in midgut 1 dpi or fat body 7 dpi (Fig. 4C). This result is consistent with the expression patterns shown by the RNA-seq data (Fig. 4A). In addition, 12 translation elements, including 4 translation initiation factors, 4 elongation factors, and 4 acidic ribosomal proteins, were also upregulated in the midgut 1 dpi or the fat body 7 dpi (Table S2). For midgut or fat body, the viral RNA abundance was less at 1 dpi or 7 dpi, respectively (Fig. S1A), which means early invasion of the virus in both tissues. These results suggest that the mosquito translational machinery was induced only during early infection.

**FIG 5:**
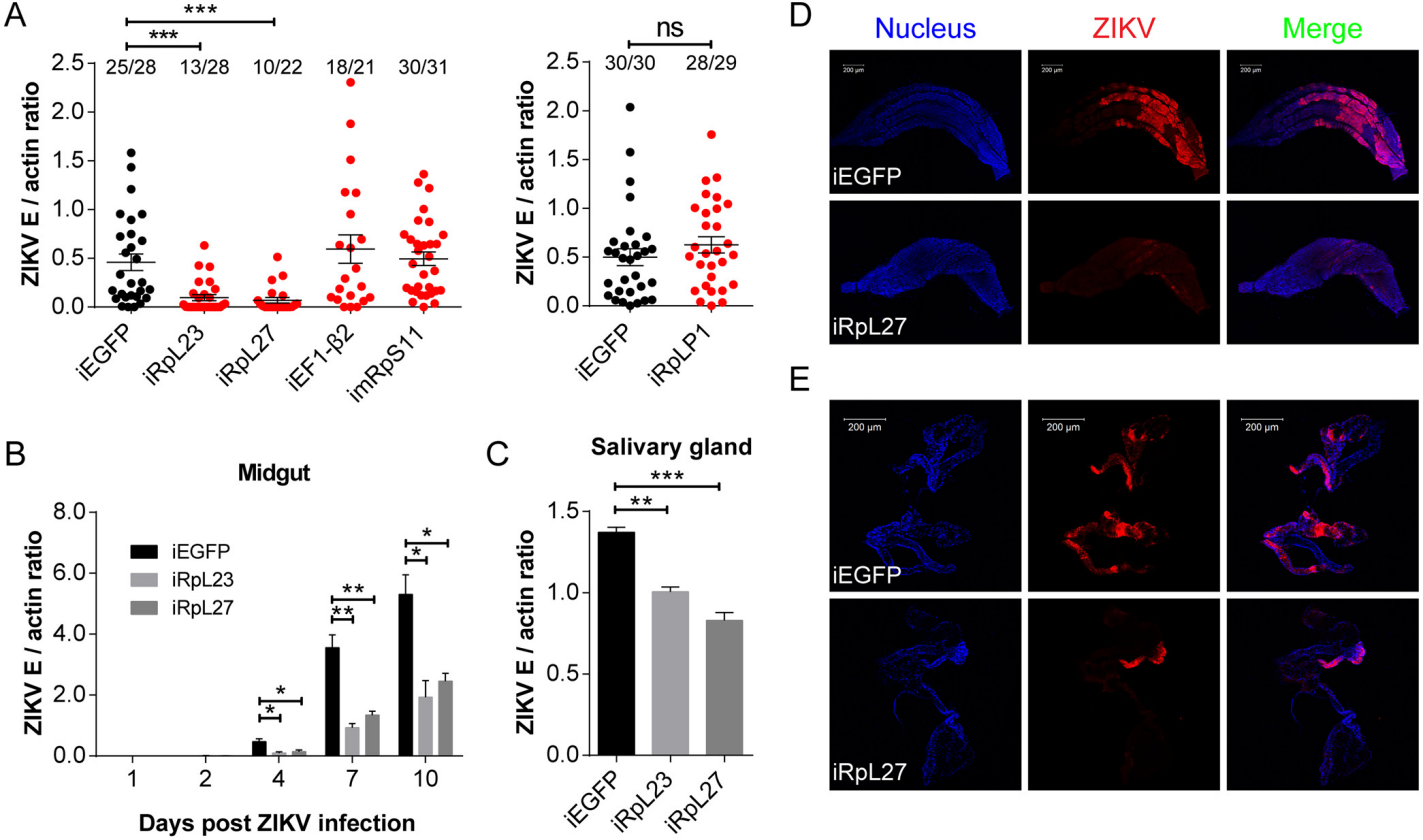
Ribosomal component proteins RpL23 and RpL27 are critical for ZIKV infection. (A to E) After gene silencing, female A. aegypti was fed blood meal containing 1.0 × 10^6^ PFU/ml ZIKV. *EGFP* dsRNA-treated mosquitoes were the control group. (A) The role of translation-related genes in ZIKV infection. At 7 dpi, the viral load of a single mosquito was tested by qPCR and normalized against the A. aegypti actin gene. The top of each column shows the ratio of the number of infected mosquitoes to the total number of mosquitoes. Data are means ± SEM. The result shown is representative of three independent experiments. *P* value was determined by a Mann-Whitney test. ***, *P < *0.001; ns, not significant. (B to E) Silencing A. aegypti *RpL23* or *RpL27* impairs ZIKV infection. (B and C) Thirty midguts (B) and 60 salivary glands (C) were pooled for viral load detection by qPCR, as mentioned above. Data are mean ± SEM from three independent experiments. Unpaired Student’s *t* tests were used for statistical analysis. *, *P < *0.05; **, *P < *0.01; ***, *P < *0.001. (D and E) At 7 dpi, midguts (D) and salivary glands (E) of mosquitoes were dissected, and the viral infection was detected by immunofluorescence assay using anti-E 4G2 antibody (red). Nuclei were stained by Hoechst 33258 (blue). The bar shows 200 μm. The result shown is representative of two independent experiments.

We then knocked down four ribosomal protein genes and one elongation factor in mosquitoes by RNAi and assessed their effect on viral load (Fig. 5A). Efficiency of RNAi knockdown was confirmed using qPCR (Fig. S4). ZIKV efficiently infected the *EGFP* dsRNA-treated mosquitoes, with a ratio nearly 90% of the total mosquitoes infected, while 46% and 45% were infected in *RpL23* and *RpL27* dsRNA-treated mosquitoes (Fig. 5A). However, three of these genes, *EF1-β2*, *mRpS11*, and *RpLP1*, did not show a significant reduction in viral load after silencing (Fig. 5A). Therefore, *RpL23* and *RpL27* were selected as targets for further investigation. After viral infection, the midgut and the salivary gland from *RpL23* and *RpL27* dsRNA-treated mosquitoes were dissected for virus detection by qPCR (Fig. 5B and C). Compared to the *EGFP* group, ZIKV levels in the midgut decreased by 2.7- to 4.8-fold from 4 to 10 days after infection (Fig. 5B), and those in the salivary gland decreased by 1.3- and 1.7-fold at 7 days after infection (Fig. 5C). Next, the midguts and the salivary glands were further tested for ZIKV infection by immunofluorescence, utilizing an antibody targeting the viral E protein. A total of 19 of the 23 midguts from the control group were found to be efficiently infected, while only 6 of the 27 midguts treated with *RpL27* dsRNA were infected (Fig. 5D). Most of the salivary glands from *RpL27* dsRNA-treated mosquitoes were negative for ZIKV infection compared to those from the *EGFP* group (Fig. 5E). These results indicate that the ribosomal proteins RpL23 and RpL27 are critical for ZIKV infection.

10.1128/mSystems.01190-20.4 FIG S4 RNAi efficiency of the translation-related genes at 7 dpi (10 days after dsRNA treatment). The gene expression was tested by qPCR and normalized against A. aegypti actin. *EGFP* dsRNA-treated mosquitoes were the control group. Data are means ± SEM (*n* ≥ 3). Download FIG S4, TIF file, 0.2 MB. Copyright © 2021 Shi et al. 2021 Shi et al. https://creativecommons.org/licenses/by/4.0/ This content is distributed under the terms of the Creative Commons Attribution 4.0 International license.

### *RpL23* and *RpL27* are transcriptionally regulated by the JH-receptor complex.

**FIG 6:**
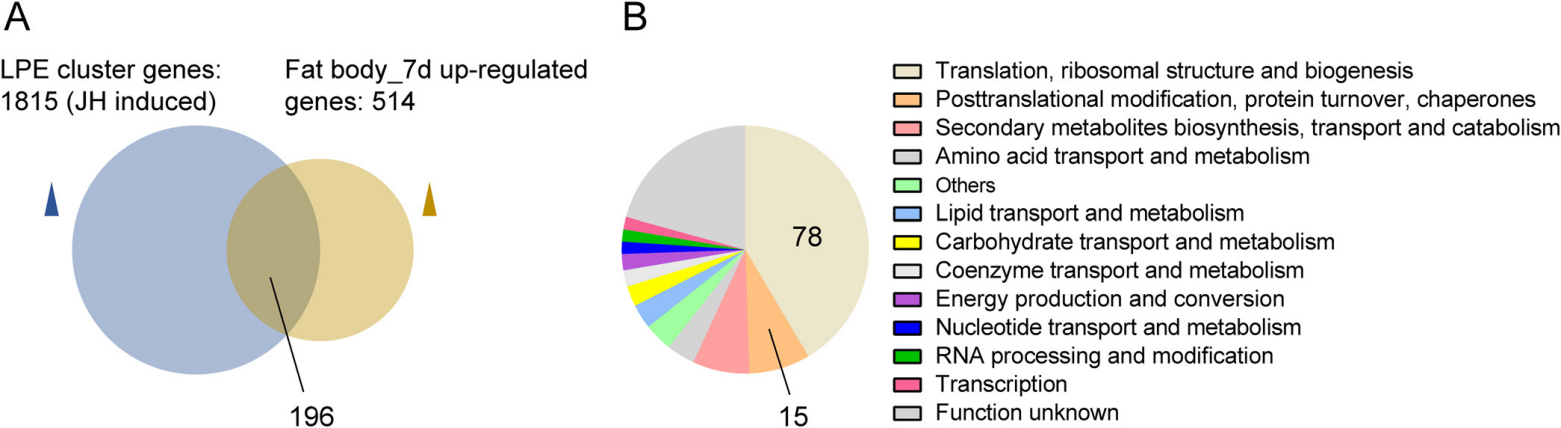
Ribosomal protein genes are potentially responsive to juvenile hormones. (A) Venn diagram comparing the upregulated genes in 7-dpi fat body transcriptome with the LPE gene cluster. LPE, late posteclosion. (B) Functional classification of 196 shared genes from panel A using the inNOG database.

We previously identified 1,815 differentially expressed transcripts potentially controlled by JH in the fat body of female A. aegypti at the late posteclosion (LPE) phase (48). Comparison of the fat body 7-dpi gene transcripts with the LPE gene cluster showed a large overlap between these transcriptomes (a total of 196) (Fig. 6A). Using the inNOG database to analyze the functional groups of these overlapping genes, 47% of the repertoire is involved in the protein translation process (78 ribosomal structures, 15 posttranslational modifications) (Fig. 6B). In addition, after viral infection, the expression of juvenile hormone acid methyltransferase (AAEL00628), a terminal enzyme that catalyzes JH biosynthesis, increased at 7 dpi in the head, while the expression of juvenile hormone epoxide hydrolase (AAEL011314), an enzyme that catalyzes JH hydrolysis, decreased at 7 dpi in the fat body (Fig. S5). These results suggest that the expression of ribosomal component proteins is responsive to JH.

10.1128/mSystems.01190-20.5 FIG S5 Gene expression involved in JH biosynthesis and degradation pathway after ZIKV infection. (A and B) The expression of *mfe* and *JHAMT* in head at 7 dpi. mfe, methyl farnesoate epoxidase; JHAMT, juvenile hormone acid methyltransferase. (C and D) The expression of JHEH genes in fat body at 7 dpi. JHEH, juvenile hormone epoxide hydrolase. (A to D) Data are means ± SEM (*n* ≥ 3). *P* value was determined by unpaired Student’s *t* tests. *, *P < *0.05. Download FIG S5, TIF file, 0.5 MB. Copyright © 2021 Shi et al. 2021 Shi et al. https://creativecommons.org/licenses/by/4.0/ This content is distributed under the terms of the Creative Commons Attribution 4.0 International license.

**FIG 7:**
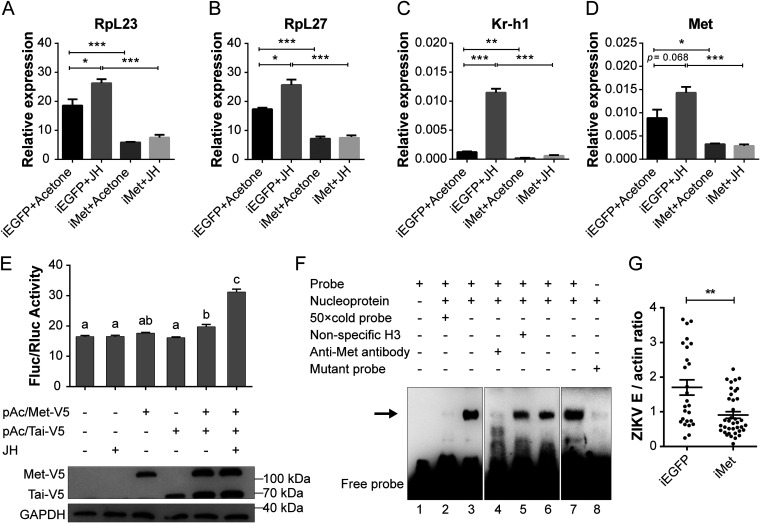
JH-receptor complex regulates the transcription of *RpL23* and *RpL27*. (A to D) Effects of JH and Met RNAi knockdown (iMet) on the expression of *RpL23* and *RpL27* in cultured fat bodies *in vitro*. The fat body isolated from female A. aegypti was cultured for 8 h under different treatment conditions. Kr-h1 (AAEL002390) was the positive control. JH, medium containing 10 μM JH; acetone, medium containing acetone. The expression level was normalized against the A. aegypti actin gene. The result shown is the mean ± SEM from three biological replicates. Unpaired Student’s *t* tests were used for statistical analysis. *, *P < *0.05; **, *P < *0.01; ***, *P < *0.001. (E) Dual luciferase reporter assay. Aag2 cells were cotransfected with pGL4.10/*RpL23* −2287 to −721, together with either a pAc5.1b empty vector or an expression vector for pAc5.1/Met-V5 or pAc5.1/Tai-V5 as indicated. At 42 h posttransfection, 20 μM JH was added to the wells as indicated. The expression of Met-V5 and/or Tai-V5 in Aag2 cells was confirmed by Western blots. GAPDH was the loading control. The columns with the letters a, b, and c show significantly different groups (*P < *0.05, one-way ANOVA). (F) EMSA confirmed the binding of JH-Met-Tai complex to the *RpL23* probe. The DNA probe (5′-CTCAAAGGAACACGCGATTGGAGGCT-3′) contains the Met putative binding motif from the *RpL23* promoter region. Unlabeled probe (50× cold probe) identified the binding specificity, and anti-Met antibody confirmed the presence of Met protein. The DNA-protein complex disappeared after the E-box-like motif (CACGCG) was mutated to TCAATA. Assays were tested with fat body nuclear extract of female A. aegypti at 72 h posteclosion. The arrow indicates the specific DNA-protein complex. (G) Depletion of Met impaired ZIKV infection. Female A. aegypti was fed blood meal containing 1.0 × 10^6^ PFU/ml ZIKV. At 7 dpi, the viral load of a single mosquito was tested by qPCR and normalized with the A. aegypti actin. iEGFP, EGFP RNAi knockdown; iMet, Met RNAi knockdown. Data are mean ± SEM. The result shown is representative of three independent experiments, and *P* value was determined by a Mann-Whitney test. **, *P < *0.01.

To determine whether JH is involved in the regulation of ribosomal protein-related gene expression, we employed an *in vitro* fat body culture assay. The expression levels of *RpL23* and *RpL27* were significantly elevated by JH but were suppressed after *Met* RNAi knockdown (iMet) (Fig. 7A and B). This result suggests that the expression of *RpL23* and *RpL27* is regulated by the JH signaling pathway. Krüppel homolog 1 (Kr-h1) has been identified as the downstream gene directly regulated by Met (49). The expression of *Kr-h1*, which served as a control, was induced by JH but inhibited by iMet (Fig. 7C). The efficiency of *Met* RNAi knockdown was confirmed using qPCR (Fig. 7D).

To understand the regulation of the expression of *RpL23* and *RpL27* by the JH signaling pathway, we examined whether Met interacts with the promoters of these genes. The putative Met-binding motif is a conserved E-box-like motif with the consensus sequence CACGYG (48). Two and one putative Met-binding motifs were found in the promoters of *RpL23* and *RpL27*, respectively (Fig. S6A). A luciferase reporter assay was then conducted to determine whether Met directly regulates *RpL23* and *RpL27* transcription. The upstream promoter sequences of *RpL23* (nucleotides −2287 to −721) and *RpL27* (nucleotides −233 to +24) were subcloned upstream of a luciferase gene. In the presence of JH, mosquito Met and Taiman (Tai) form a heterodimer and then activate the transcription of JH-regulated genes (50). The expression of Met, Tai, or Met plus Tai was validated by Western blotting with anti-V5 antibodies (Fig. 7E, Fig. S6B). Obviously, after the addition of JH, coexpression of Met and Tai strikingly upregulated the luciferase activities, but no protein expression or expression of either Met or Tai alone in Aag2 cells could increase the luciferase activities (Fig. 7E, Fig. S6B), which indicates that *RpL23* and *RpL27* transcription requires the JH-Met-Tai complex.

10.1128/mSystems.01190-20.6 FIG S6 (A) Schematic diagram of Met putative binding motifs of *RpL23* and *RpL27*. Two and one E-box-like motifs (CACGYG) were identified from the promoter regions of the *RpL23* and *RpL27*, respectively. The E-box-like motif is shown in the green box. (B) Luciferase reporter assays. Aag2 cells were cotransfected with pGL4.10/RpL27 −233 to +24, together with either a pAc5.1b empty vector or an expression vector for pAc5.1/Met-V5 or pAc5.1/Tai-V5, as indicated. At 42 h posttransfection, 20 μM JH was added to the wells as indicated. The expression of Met-V5 and/or Tai-V5 in Aag2 cells was confirmed by Western blots. GAPDH was the loading control. The columns with the letters a and b show significantly different groups (*P < *0.05, one-way ANOVA). (C) EMSA confirmed the binding of JH-Met-Tai complex to the *RpL27* probe. Unlabeled probe (50× cold probe) identified the binding specificity, and anti-Met antibody confirmed the presence of Met protein. The DNA-protein complex disappeared after the E-box-like motif (CACGCG) was mutated to TCAATA. Assays were tested with fat body nuclear extract of female A. aegypti at 72 h posteclosion. The arrow indicates the specific DNA-protein complex. Download FIG S6, TIF file, 1.3 MB. Copyright © 2021 Shi et al. 2021 Shi et al. https://creativecommons.org/licenses/by/4.0/ This content is distributed under the terms of the Creative Commons Attribution 4.0 International license.

Next, electrophoretic mobility shift assays (EMSAs) were performed to determine the physical interaction between the JH-receptor complex and the *RpL23* and *RpL27* promoters. A band shift was visualized in samples incubated with the labeled *RpL23* probe (lanes 3, 6, and 7) or *RpL27* probe (lanes 3 and 4) (Fig. 7F, Fig. S6C). This disappeared after preincubation with unlabeled *RpL23* or *RpL27* probe (both lane 2), indicating that the DNA-protein interaction is specific. Preincubation of the anti-Met antibody with nuclear extract resulted in the loss of mobility bands (Fig. 7F, Fig. S6C), suggesting Met binds specifically to the promoter regions. The band shift was abolished after mutation (Fig. 7F, Fig. S6C), suggesting this motif is crucial for interaction. These data demonstrate that JH-receptor complex can transcriptionally regulate the expression of these genes.

### Met RNAi depletion suppressed ZIKV infection in A. aegypti.

Ribosomal proteins RpL23 and RpL27 are critical for ZIKV infection, and they can be directly regulated by the JH-receptor complex. To determine whether the JH signaling pathway also affects the viral infection of A. aegypti, an *in vivo* hormone application and RNAi assay were carried out. In nature, viruses infect mosquitoes through a blood meal. The JH titer is very low at 6 h postblood meal (PBM), elevated by 36 to 72 h PBM, and maintained at a high level until the next blood meal (51). Thus, to study the effect of JH on viral infection, we first fed mosquitoes with ZIKV-infected blood, topically applied 1 ng JH, in acetone, to the abdomen of female mosquitoes (6 h PBM), and tested the viral load by qPCR at 48 h after infection. The viral load was not enhanced after the addition of JH compared to the control (Fig. S7A). There may be an antagonistic effect of 20E on JH during the PBM phase, or the treatment was insufficient to obtain this phenotype, because the expression of *RpL23* and *RpL27* only increased by 0.2-fold after adding JH (Fig. S7B and C). However, the *Met* RNAi depletion resulted in a significant reduction in ZIKV genome levels compared with those in mosquitoes treated with *EGFP* dsRNA (Fig. 7G, Fig. S7D). The expression of *RpL23* and *RpL27* was significantly suppressed in iMet (Fig. S7E, Fig. S7D). To confirm whether the iMet-reduced infection is direct or mediated by an intermediate factor, two transcription factors (Kr-h1 and Hairy), directly regulated by Met, were used to evaluate the viral infection on mosquitoes. Suppression of *Kr-h1* and *Hairy* in iMet was confirmed using qPCR (Fig. S7G, Fig. S7H). iKr-h1 or iHairy had no effect on virus titer (Fig. S7I), suggesting that iMet reduces infection by inhibiting the transcription of ribosomal protein genes. It is noteworthy that iRpL23 and iRpL27 reduced the titer and infection rate of ZIKV in mosquitoes, but iMet only inhibited the virus titer in mosquitoes (Fig. 5A and 7G). These data demonstrate that the expression of ribosomal protein genes is partially dependent on JH.

10.1128/mSystems.01190-20.7 FIG S7 (A) Effect of JH on the ZIKV infection. Female A. aegypti was fed blood meal containing 1.0 × 10^6^ PFU/m ZIKV. Acetone or acetone with 10 μM JH then was added onto the abdomen of females at 6 h PBM. At 48 h postinfection, the viral load of a single mosquito was tested by qPCR and normalized against the A. aegypti gene actin. The top of each column shows the ratio of the number of infected mosquitoes to the total number of mosquitoes. Data are means ± SEM. The experiment was repeated twice with similar results. A Mann-Whitney test was used for the statistical analysis. ns, not significant. (B and C) The expression of *RpL23* and *RpL27* after adding JH. NT, females without any treatment. Data are means ± SEM (*n* ≥ 3). *P* value was determined by unpaired Student’s *t* tests. *, *P < *0.05; **, *P < *0.01. (D) RNAi efficiency of Met. (E to H) Effects of Met RNAi knockdown on the expression of *RpL23*, *RpL27*, *Hairy*, and *Kr-h1*. (D to H) At 3 days after dsRNA treatment, the gene expression was tested by qPCR and calculated against the A. aegypti actin gene. iEGFP, EGFP RNAi knockdown; iMet, Met RNAi knockdown. Data are means ± SEM (*n* = 3). *P* value was determined by unpaired Student’s *t* tests. **, *P < *0.01; ***, *P < *0.001. (I) Depletion of Hairy or Kr-h1 does not affect ZIKV infection. Female A. aegypti was fed blood meal containing 1.0 × 10^6^ PFU/m ZIKV. At 7 dpi, the viral load of a single mosquito was tested by qPCR and normalized against the A. aegypti actin gene. The number of mosquitoes used for testing is shown in brackets. Data are means ± SEM. The result shown is pooled from two independent experiments. A Mann-Whitney test was used for the statistical analysis. ns, not significant. Download FIG S7, TIF file, 0.7 MB. Copyright © 2021 Shi et al. 2021 Shi et al. https://creativecommons.org/licenses/by/4.0/ This content is distributed under the terms of the Creative Commons Attribution 4.0 International license.

## DISCUSSION

**FIG 8:**
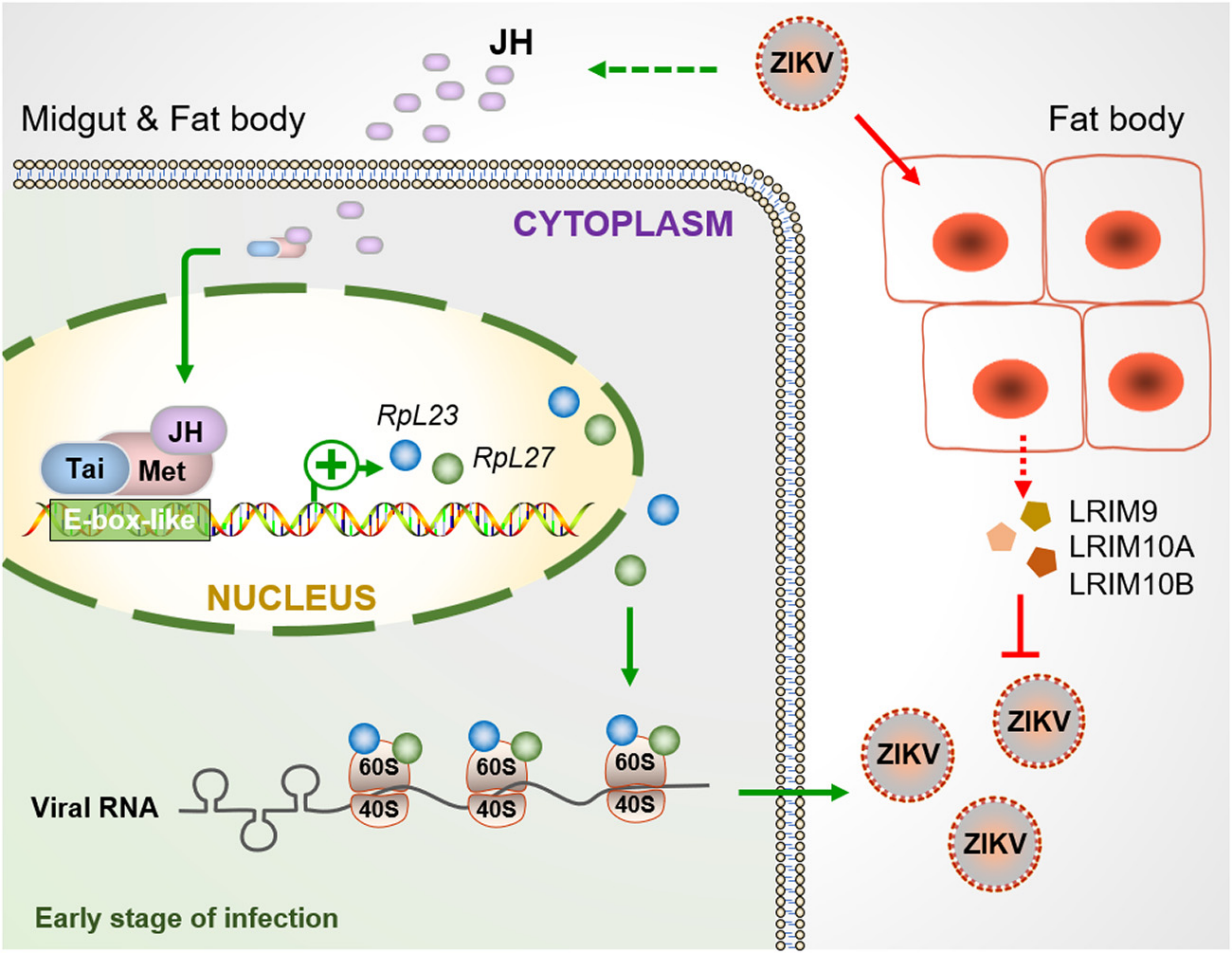
Model for the function of ribosomal protein and LRIMs during ZIKV infection. At early stages of infection, ZIKV regulates many ribosomal protein genes (including *RpL23* and *RpL27*) through JH signal. JH-Met-Tai acts on the promoters of *RpL23* and *RpL27* to activate their transcription, which facilitates the translation of viral proteins and promotes viral infection. Moreover, ZIKV induces fat body to secrete LRIMs, including LRIM9, LRIM10A, and LRIM10B, which act as antagonists to inhibit infection.

Pathogens transmitted by mosquitoes have a great impact on human health. For example, ZIKV is an emerging virus that has caused epidemics of neurological diseases such as Guillain–Barré syndrome and microcephaly in the Americas during 2015 to 2016 (2, 3). The interactions of mosquitoes and other viruses have been extensively studied, including antiviral immune mechanisms (12). However, the interaction mechanism between ZIKV and A. aegypti has not been studied. We used transcriptomic and genetic technology to identify several virus restriction and dependency factors in A. aegypti. Several LRIM proteins with leucine-rich repeats had moderate antiviral responses during mosquito infection (Fig. 8). The viral infection activated the global ribosome activities regulated by the JH-receptor complex, thereby promoting infection (Fig. 8). Inhibiting juvenile hormone signals effectively blocked viral infection by downregulating ribosomal protein expression. Therefore, our data provide valuable evidence for understanding the transmission mechanism of the pathogens in vector hosts.

Insects lack adaptive immune systems. They rely on inherent antiviral mechanisms in cells, such as RNAi and innate immune responses, to limit the propagation of viruses. Previous studies have provided evidence that immune signaling pathways, such as Toll, IMD, and JAK-STAT, have antiviral roles in D. melanogaster (29, 52, 53). In mosquitoes, these pathways also have defensive roles against DENV and WNV (28, 30, 33, 34). However, inhibition or activation of these pathways did not affect the ZIKV burden in A. aegypti. Our results suggest that ZIKV evolved escape mechanisms to resist these defense responses. Antiviral RNAi is an intrinsic immune defense mechanism conserved in insects, and flaviviruses can induce vsiRNA production in mosquito cells (24, 25, 54). Although vsiRNA transgenic mosquitoes can effectively block virus transmission (55, 56), downregulation of *Dicer2* and *Ago2* did not enhance ZIKV infection in mosquitoes. Owing to the high laboratory mosquito infection rate (>90%) with ZIKV, we speculate that the viral load in susceptible A. aegypti strains already is operating at maximum capacity. Thus, the role of antiviral RNAi may be hidden or antagonized by the virus. Interestingly, some flaviviruses encode viral suppressors of RNAi (VSR) as immune evasion strategies. The sfRNA (subgenomic flavivirus RNA) has recently been reported as a potential VSR, which determines ZIKV transmission in mosquitoes (57). Yang et al. demonstrated that the nonstructural protein NS2A of DENV2 acts as a VSR during viral infection in both mammalian and mosquito cells (43).

Although the Toll, IMD, JAK-STAT, and RNAi pathways might not be required for anti-ZIKV defense, other immune systems, such as ROS, can still effectively protect against viral infections. Clearance of midgut ROS by induction of HPX8C is a determinant of DENV and ZIKV infection (41). Nonstructural protein NS1 and host serum iron modulating DENV acquisition by mosquitoes is also associated with ROS (14, 58). Therefore, to identify additional antiviral effectors, we examined immunity-related gene expression profiles after ZIKV infection of A. aegypti. Viral infection caused a moderate immune response. After ZIKV infection, a cohort of recognition factors, signal cascade molecules, and effectors were upregulated. These immune molecules contained some *CTLs*, *PGRPs*, *AMPs*, and ROS-related genes, most of which have been shown to be defensive against pathogens (31, 41, 42, 59, 60). In addition, serine protease cascades are involved in numerous immune responses, such as Toll pathway activation and melanization. An important component of these cascades is CLIP-SP and their specific inhibitors, SRPN, which are essential for activating PPO during infections. Some of these, including CLIPB8, CLIPB9, CLIPB5, CLIPA28, SRPN1, and SRPN2, are involved in mosquito reactions against malaria and fungi (61, 62). We found that many CLIP-SPs and SRPNs, as well as two PPOs (PPO3 and PPO5), were induced by ZIKV infection in A. aegypti. These results suggest that melanization response is involved in the antiviral defense of mosquitoes. The melanization response, also a robust antiviral defense of Helicoverpa armigera against baculovirus, has been studied (63), but its antiviral role in mosquitoes has not been confirmed. Leucine-rich repeat proteins LRIM1, LRIM2, and LRRD7 are powerful antagonists of malaria parasite *Plasmodium* infection in mosquitoes (47, 64). LRIM9, LRIM10A, and LRIM10B also showed antiviral effects in this study, but their molecular mechanism is unclear. Although immunity-related genes were activated after infection, the response was different in the midgut and the fat body. To identify more antiviral factors in insects, it will be necessary to understand the antiviral immune response at the level of specific tissues and cell populations.

Successful viral infection requires a variety of host factors to work for the virus. Early translation of flavivirus proteins is critical for virus replication after entry. The host cellular translational machinery is necessary to achieve this goal, and the ribosome is at the center of this machinery. Flaviviruses can control host translation machinery to increase translation of viral proteins. For example, DENV and ZIKV 5′ untranslated regions function as internal ribosome entry sites to initiate viral protein translation (65). DENV can uncouple the translational inhibition caused by cellular stress by dephosphorylation of eIF2α (translation initiation factor 2 alpha) (66, 67). Additionally, the acidic ribosomal protein RpLP1/2 complex is required for translation of flavivirus RNA in mammal cells or mosquitoes (20). In the present study, 73% of ribosomal protein genes in A. aegypti were upregulated after ZIKV infection, and silencing of *RpL23* and *RpL27* significantly attenuated ZIKV infection. However, RpLP1 did not appear to be required for ZIKV protein translation in A. aegypti. Finally, we found that ZIKV enhanced ribosomal activity through the JH-receptor complex during infection and blocking JH signals reduced the infection level, because depletion of Met can also block the reproductive development of mosquitoes (68). Therefore, although PPF and DBH are considered suitable alternatives to traditional chemical pesticides, our findings also suggest blocking the transmission of ZIKV between mosquitoes and humans using JH antagonists.

## MATERIALS AND METHODS

### Ethics statement.

The blood for feeding mosquito was collected from healthy mice raised in an animal biosafety laboratory level 3 (A-BSL3). All ZIKV and mosquito experiments were conducted under biosafety level 2 (BSL2) and approved by the Bioethics Committee of the Institute of Zoology, Chinese Academy of Science.

### Mosquitoes, cells, and viruses.

A. aegypti (UGAL/Rockefeller strain) mosquitoes were maintained in the laboratory as described previously (69, 70). The ZIKV Natal-RGN strain (GenBank accession number KU527068) was passaged in C6/36 cells for mosquito oral infection. The C6/36 cells were kept in RPMI 1640 medium containing 8% heat-inactivated fetal bovine serum (FBS; Gibco) at 28°C and 5% (vol/vol) CO_2_. The virus titers were determined by a plaque formation assay as described previously (42). The Aag2 cells used for the dual luciferase reporter assay were kept in Schneider *Drosophila* medium containing 8% FBS at 28°C.

### Viral infection of mosquitoes.

Females aged 3 to 4 days old were used for oral infection experiments. Viruses were diluted with mouse blood or RPMI 1640 medium at a ratio of 1:1 and preheated at 37°C for 30 min before feeding. The mixture containing 1.0 × 10^6^ PFU/ml ZIKV then was fed to mosquitoes through a thin Parafilm at 37°C for 30 min. The mosquitoes were then anesthetized at 4°C for 10 min, and those with a full blood meal were selected for further experiments.

### Library preparations and Illumina sequencing.

For RNA-seq analysis, mosquitoes were fed on ZIKV-infected blood or blood without virus (mock). At 1 or 7 dpi, the midguts and fat bodies from 30 individual mosquitoes were dissected in phosphate-buffered saline (PBS). Total RNA from midguts or fat bodies was extracted with the TRIzol kit (Invitrogen). The libraries were constructed at the Novogene Bioinformatics Technology Co., Ltd. (Beijing, China) and sequenced on the Illumina HiSeq 2000 platform.

### Bioinformatics analysis.

The high-quality clean reads were filtered from the raw reads by removing the adaptor sequences and low-quality sequences with ambiguous N nucleotides. The clean reads then were mapped to the reference genome using HISAT (2.0.4) with default parameters (71). The A. aegypti genome (accession ID AaegL3.4) from the VectorBase database was used for annotation. The number of reads mapped to each gene was determined using HTseq (v0.6.1), and the gene expression levels were represented by the expected number of fragments per kilobase of transcript sequence per millions of base pairs sequenced (FPKM). DEG analysis was implemented through the edgeR (3.0.8) package in the R environment (72). The genes with FPKM values of less than 0.5 in the infected and mock group were filtered to avoid unreliable fold changes caused by random noise related to low signal values. The genes with a *P* value of less than 0.01 and a fold change of greater than 1.5 were considered differentially expressed. Annotation of the immune gene was assigned based on immuneDB (45). GO enrichment analysis of upregulated and downregulated genes was implemented by the GOseq (Release 2.12) package in the R environment (73). The KOBAS 2.0 web server was used for KEGG pathway enrichment analysis (74). GO terms and pathways with a corrected *P* value (equivalent to the false discovery rate [FDR] value) of less than 0.05 were considered significantly enriched.

### RNA extraction and qPCR analysis.

Total RNA samples from whole mosquito bodies or pooled tissues were extracted with a TRIzol kit. A 1-μg sample of total RNA was used for synthesis of first-strand cDNA using a PrimeScript RT reagent kit (TaKaRa). qPCR was performed using a SuperReal premix plus (Tiangen) on the Applied Biosystems Step-One Plus system. The cycling parameters were 95°C for 3 min for initial denaturation, followed by 40 cycles at 95°C for 10 s and 60°C for 30 s. A melting curve analysis at 60°C to 95°C was performed to ensure that only a single product was amplified. The expression level was normalized against the A. aegypti actin gene (AAEL011197) for each sample. Primers are listed in Table S3 in the supplemental material.

10.1128/mSystems.01190-20.10 TABLE S3 Primers used in this work. Download Table S3, XLSX file, 0.01 MB. Copyright © 2021 Shi et al. 2021 Shi et al. https://creativecommons.org/licenses/by/4.0/ This content is distributed under the terms of the Creative Commons Attribution 4.0 International license.

### Detection of viral load.

For the detection of a single mosquito virus load, total RNA was extracted with a TRIzol kit. A 20-ng sample of total RNA was directly used for qPCR analysis using a one-step TB green PrimerScript RT-PCR kit (TaKaRa). qPCR was performed on the Applied Biosystems Step-One Plus system with reverse transcription at 42°C for 5 min and then 40 cycles of 95°C for 5 s and 60°C for 30 s. A melting curve analysis were done by following the operation of the instrument. The viral RNA load was normalized to the A. aegypti actin gene, and relevant primers are shown in Table S3.

### Synthesis and microinjection of double-stranded RNA.

Double-stranded RNA (dsRNA) was prepared using the T7 RiboMAX express RNAi system (Promega), and then 1.2 μg per 207 nl of dsRNA was microinjected into the thorax of 1-day-old female mosquitoes using a Nanoliter 2000 injector (World Precision Instruments). Control mosquitoes were injected with the same amount of *EGFP* (enhanced green fluorescent protein) dsRNA. After a 3-day recovery period, the dsRNA-treated mosquitoes were used for further experiments. The efficiency of RNAi knockdown was determined by qPCR. The relevant primers are listed in Table S3.

### Immunofluorescence staining and confocal microscopy.

The midguts and salivary glands from virus-infected mosquitoes were dissected in PBS. The dissected samples were placed on chamber slides and fixed with 4% paraformaldehyde for 30 min at room temperature. After permeating with 0.5% Triton X-100 for 10 min at room temperature, the tissues were blocked in 3% bovine serum albumin (BSA). Subsequently, they were incubated with antibody 4G2 overnight at 4°C and Alexa Fluor 546 goat anti-mouse secondary antibody (1:400) for 1 h at room temperature. The 4G2 is a mouse monoclonal antibody against all flaviviruses E proteins (42). Hoechst 33258 (Invitrogen) was added at a concentration of 1 μg/ml to stain the nucleus. Images were acquired using a confocal microscope (Zeiss LSM 710; Germany).

### *In vivo* hormone application and *in vitro* fat body culture.

JH (Sigma) dissolved in acetone was used for *in vivo* hormone application assays. About 1 ng JH was applied locally to the abdomens of female mosquitoes (6 h PBM). Control samples were treated with acetone without JH. Fat body culture assays were conducted using a previously described method (70). Fat bodies dissected from *EGFP* or *Met* dsRNA-injected female mosquitoes were cultured in a medium containing 10 μM JH or acetone alone for 8 h. After incubation, samples were harvested for qPCR analysis.

### Dual luciferase reporter assay and Western blotting.

The DNA sequences coding A. aegypti Met and Tai were amplified and subcloned into a pAc5.1/V5/His expression vector (Invitrogen) fused with V5. The promoter region of *RpL23* (nucleotides −2287 to −721) and *RpL27* (nucleotides −233 to +24) was amplified and subcloned into the reporter vector pGL4.10 (Promega). The relevant primers are listed in Table S3. Afterward, 300 ng of pGL4.10 recombinant plasmid and 30 ng of pGL4.73 control plasmid containing *Renilla* luciferase gene were cotransfected into Aag2 cells. Transient transfections were carried out using FuGENE 6 transfection reagent (Promega). Some wells were also cotransfected with pAc5.1/Met-V5, pAc5.1/Tai-V5, or both. After 42 h, 20 μM JH was added to the wells for 8 h. The luciferase activity was examined using the Dual-Luciferase reporter assay system and a GloMax 96 microplate luminometer (Promega). Total protein extracts from Aag2 cells were used for Western blots described previously (70). Mouse anti-V5 antibodies (Invitrogen) were used to detect Met-V5 and Tai-V5 fusion proteins. Polyclonal antibodies against glyceraldehyde-3-phosphate dehydrogenase (GAPDH; Easybio) were used as a loading control.

### EMSA.

The fat bodies nuclear protein of female mosquitoes 72 h PE was extracted using the NE-PER nuclear and cytoplasmic extraction reagents kit (Pierce). EMSA was performed using the chemiluminescent nucleic acid detection module kit (Pierce). Biotin-labeled *RpL23* or *RpL27* probes were incubated for 30 min with nuclear protein extracts in the binding buffer provided in the kit. In the competition assays, a 50-fold amount of unlabeled probe (50× cold probe) was added into the binding reaction. In the supershift studies, the rabbit polyclonal antibody against Met or the mouse monoclonal anti-histone H3 antibodies (BE3015-100; Easybio) were preincubated with nuclear extracts before mixing with labeled probe. DNA-protein complexes were separated in a 6% (vol/vol) native polyacrylamide gel. The bands were visualized with X-ray film. The probes and mutant probes used are shown in Table S3.

### Statistical analysis.

All statistical analyses were done in GraphPad Prism 6 statistical software. Before statistical analysis, normality and homogeneity of data variance were evaluated. For the qPCR analysis, statistical significance was performed by the unpaired Student's *t* test. Comparison of single mosquito virus load was performed using the nonparametric Mann-Whitney test. The data are shown as the means ± standard errors of the means (SEM). Differences between comparisons were considered statistically significant at a *P* value of <0.05.

### Data availability.

The sequence raw data are available in the NCBI SRA database under the BioProject accession number PRJNA668685. The data supporting the findings are included in this article and its supplemental material.
