# Supplementary figures and images for: Juvenile Hormone-Sensitive Ribosomal Activity Enhances Viral Replication in Aedes aegypti
Source: mSystems. 2021 May 26;6(3):e01190-20. doi: 10.1128/mSystems.01190-20 (PMC8269256; doi:10.1128/mSystems.01190-20)

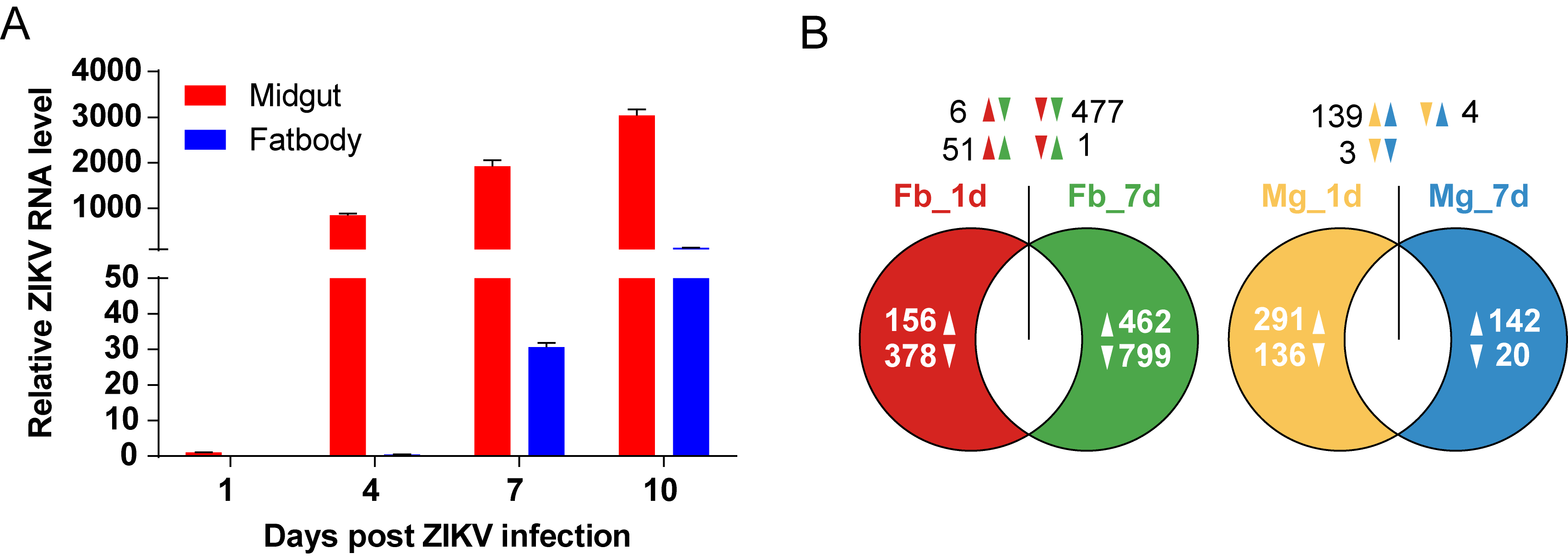

Supplement: FIG S1 [file msystems.01190-20-sf001.tif]

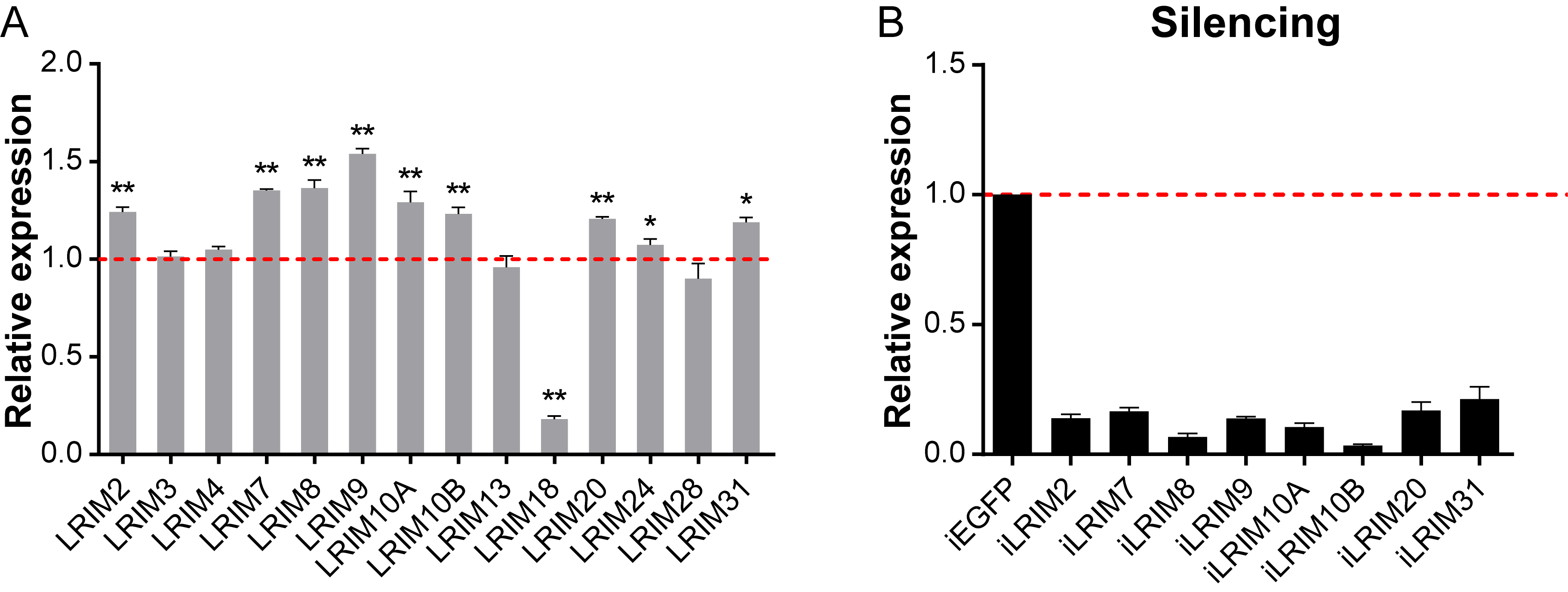

Supplement: FIG S2 [file msystems.01190-20-sf002.tif]

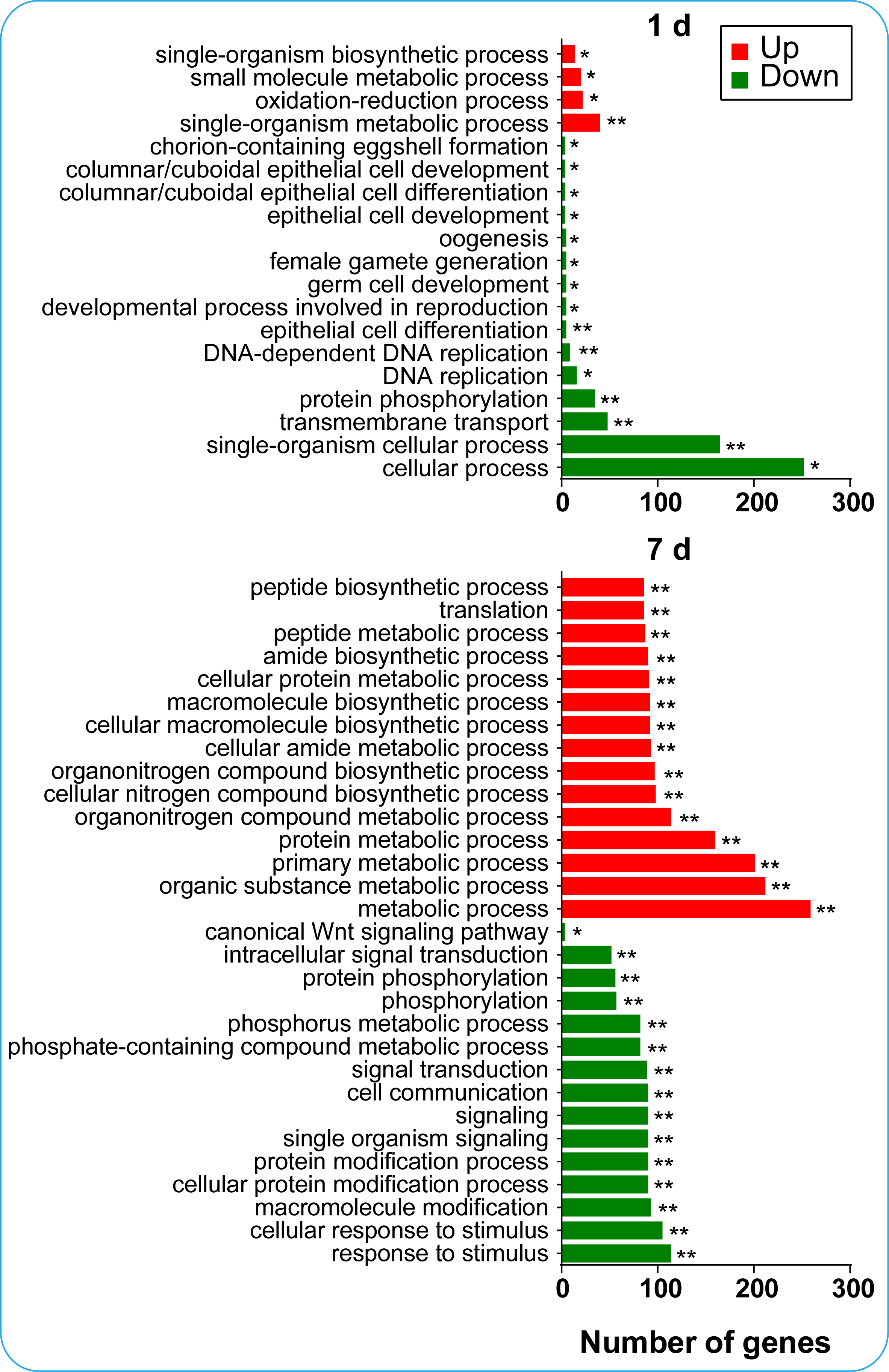

Supplement: FIG S3 [file msystems.01190-20-sf003.tif]

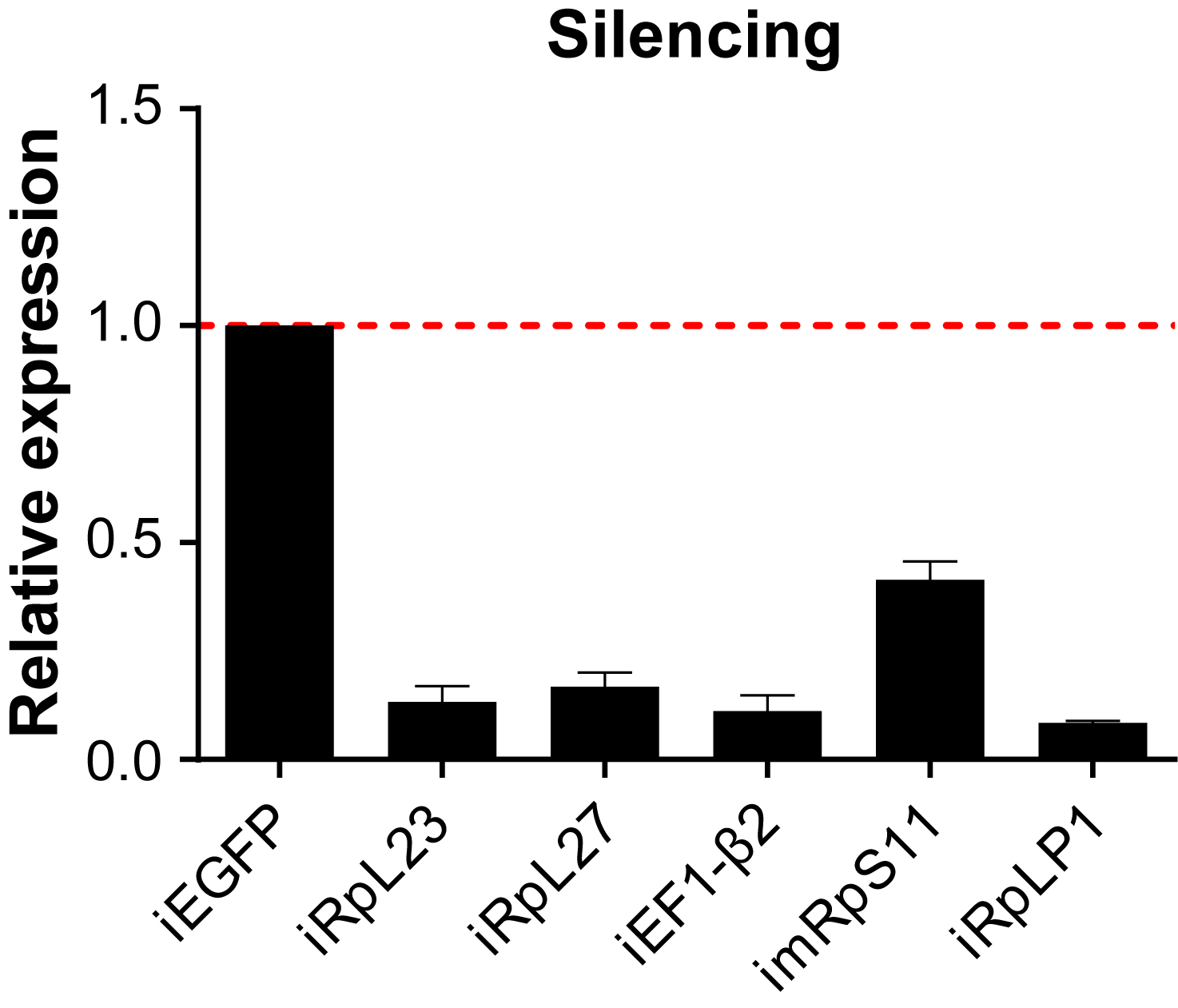

Supplement: FIG S4 [file msystems.01190-20-sf004.tif]

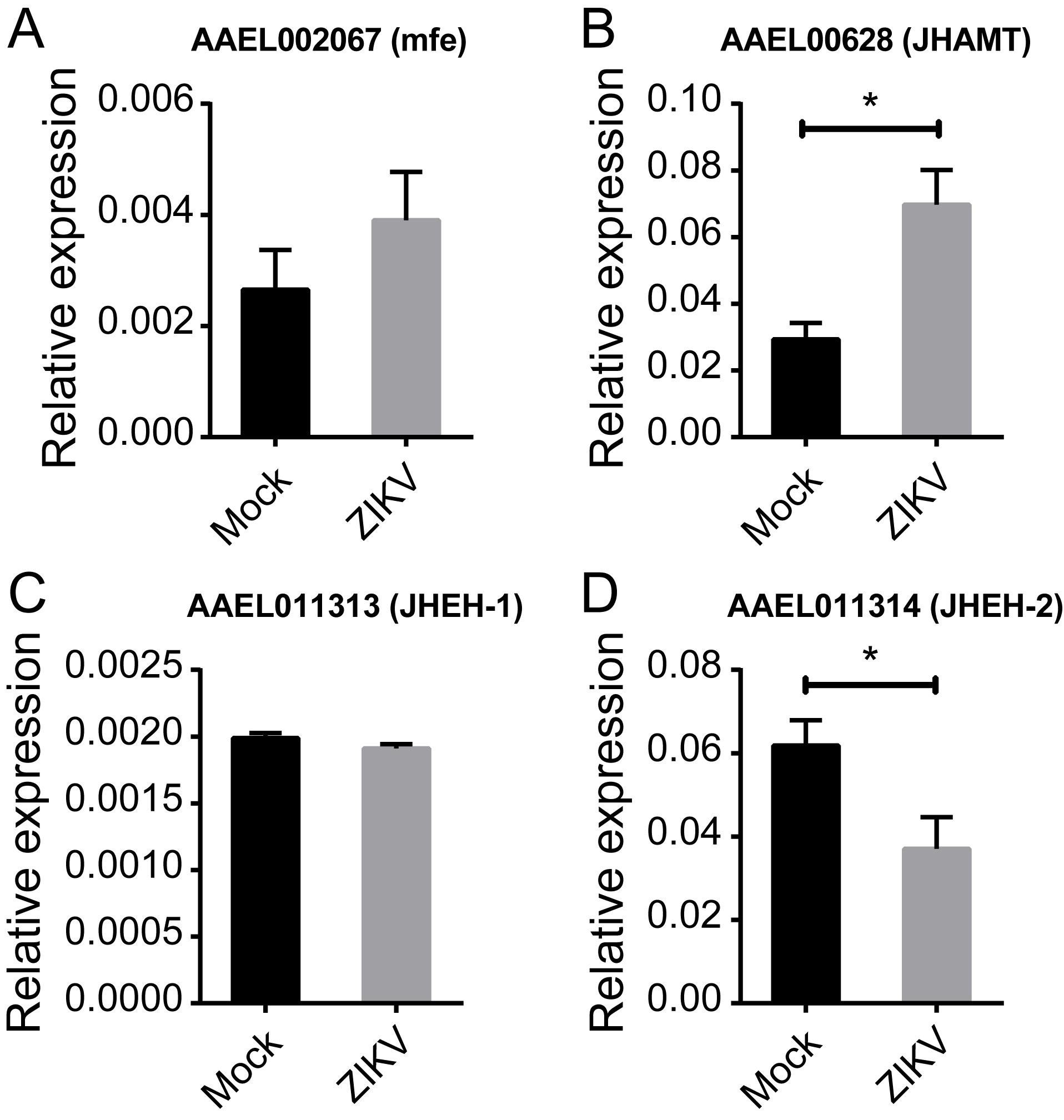

Supplement: FIG S5 [file msystems.01190-20-sf005.tif]

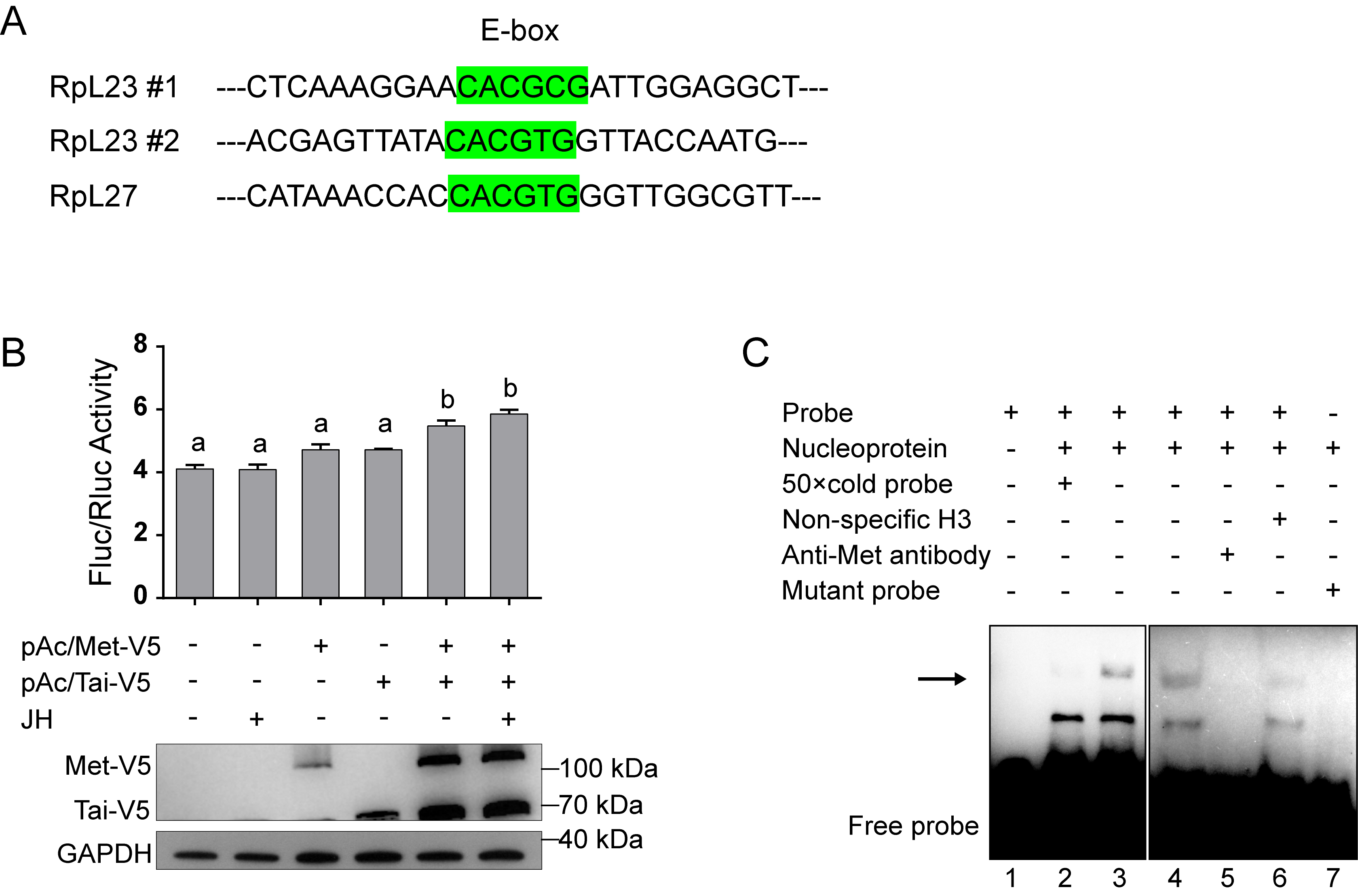

Supplement: FIG S6 [file msystems.01190-20-sf006.tif]

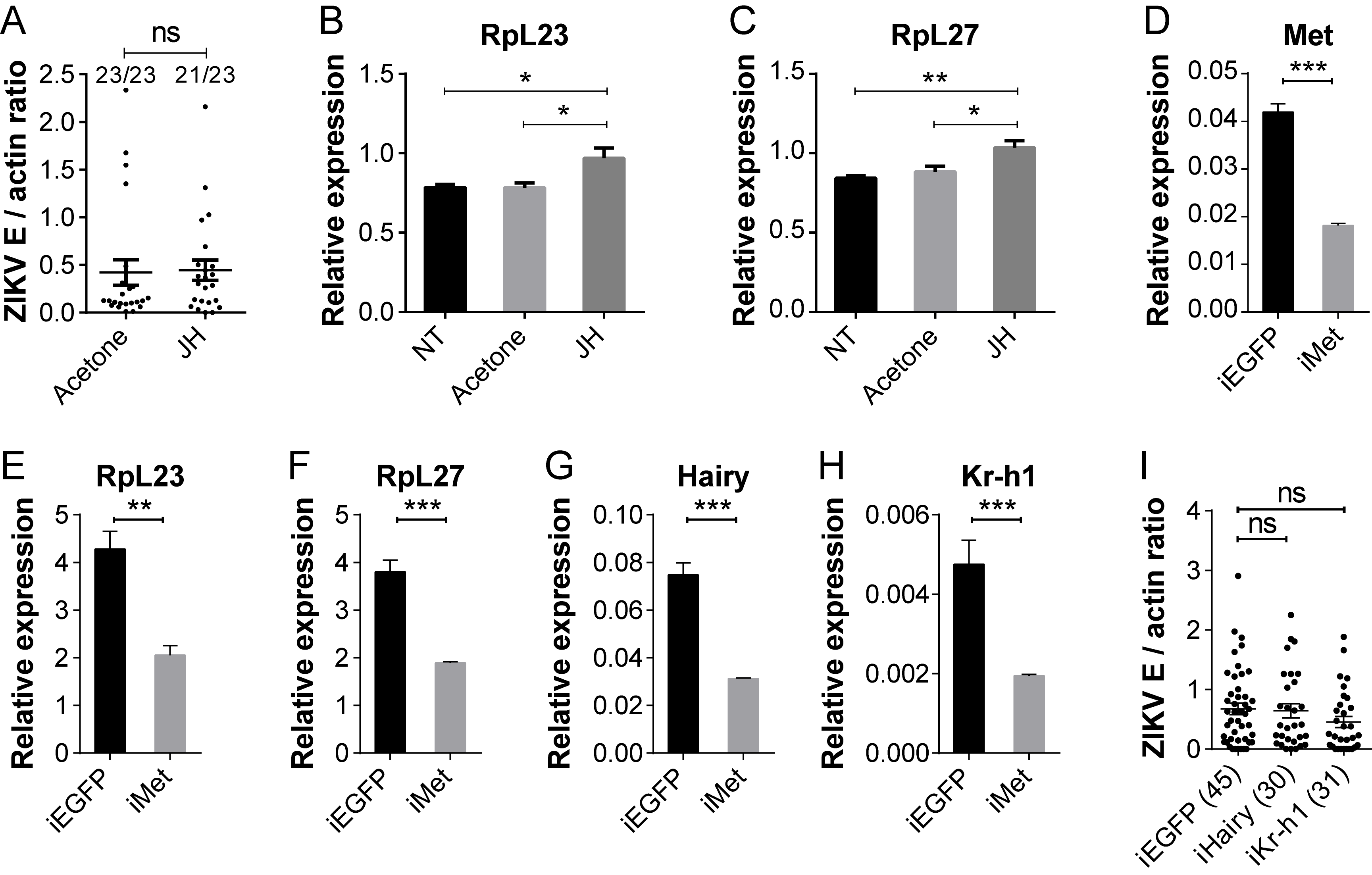

Supplement: FIG S7 [file msystems.01190-20-sf007.tif]
